# Association study of MCP-1 promoter polymorphisms with the susceptibility and progression of sepsis

**DOI:** 10.1371/journal.pone.0176781

**Published:** 2017-05-04

**Authors:** Junbing He, Yuhua Chen, Yao Lin, Wenying Zhang, Yujie Cai, Feng Chen, Qinghui Liao, Zihan Yin, Yan Wang, Shoubao Tao, Xiaoli Lin, Pengru Huang, Lili Cui, Yiming Shao

**Affiliations:** 1 The Intensive Care Unit, Guangdong Key Laboratory of Age-Related Cardiac and Cerebral Diseases, Affiliated Hospital of Guangdong Medical University, Zhanjiang, Guangdong, China; 2 The Department of Endocrinology and Metabolism, Longgang District People's Hospital of Shenzhen, Shenzhen, Guangdong, China; 3 The Department of Stomatology, Jieyang Affiliated Hospital, SunYat-sen University, Jieyang, Guangdong, China; 4 Institute of Neurology, Guangdong Key Laboratory of Age-Related Cardiac and Cerebral Diseases, Affiliated Hospital of Guangdong Medical University, Zhanjiang, Guangdong, China; Katholieke Universiteit Leuven Rega Institute for Medical Research, BELGIUM

## Abstract

Previous studies have indicated that the monocyte chemo-attractant protein 1 (MCP-1), also referred to as C-C motif chemokine ligand 2 (CCL2), plays a significant role in the pathogenesis of sepsis, and this study investigated the clinical relevance of two MCP-1 gene polymorphisms on sepsis onset and progression. The Multiplex SNaPshot genotyping method was used to detect MCP-1 gene polymorphisms in the Chinese Han population (403 sepsis patients and 400 controls). MCP-1 mRNA expression levels were measured using real-time quantitative PCR, and enzyme-linked immunosorbent assays were used to analyze MCP-1, tumor necrosis factor-alpha (TNF-α), interleukin 6 (IL-6) and interleukin-1 beta (IL-1β) plasma concentrations. The rs1024611 polymorphism analysis showed lower frequencies of minor homozygous genotype (AA) and allele (A) in sepsis patients compared to the healthy controls (19.4% vs. 31.5%, P = 0.0001 and 45.9% vs. 54.8%, P = 0.0004, respectively). And the frequencies of GG genotype and G allele were lower in sepsis patients compared to the controls (19.6% vs. 31.3%, P = 0.0002 and 46.0% vs. 54.5%, P = 0.0007, respectively). The rs1024611 AG/GG and rs2857656 GC/CC genotypes were both overrepresented in patients with severe sepsis (both P = 0.0005) and septic shock (P = 0.010 and P = 0.015, respectively) compared to the patients with mild sepsis. Moreover, among sepsis patients, the rs1024611 AG/GG and rs2857656 GC/CC carriers exhibited significant increases in expression levels of MCP-1 (P = 0.025), TNF-α (P = 0.034) and IL-6 (P = 0.043) compared with the rs1024611 AA or rs2857656 GG carriers. This study provides valuable clinical evidence that the MCP-1/CCL2 polymorphisms rs1024611 and rs2857656 are associated with sepsis susceptibility and development. We conclude that MCP-1/CCL2 plays a significant role in the pathogenesis of sepsis, which has potentially important therapeutic implications.

## Introduction

Sepsis is a systemic inflammatory disease resulting from a harmful response to microbial infection [1–3]. Although the pathological mechanism of sepsis remains unclear, numerous lines of evidence have demonstrated that variations in genes associated with the inflammatory immune response play vital roles in the pathomechanism and progression of sepsis [4–7]. Progress in genetic sequencing and association studies between different immunological profiles and disease outcomes may someday allow for genetic diagnosis and interventional treatment of sepsis, ultimately improving outcomes for critically ill patients [8–10].

Monocyte chemo-attractant protein 1 (MCP-1), also known as CC motif chemokine ligand 2 (CCL2), is an important molecule for monocytes chemotaxis, endothelial activation and regulation of leukocyte function, mediating a variety of inflammation-promoting biological activities [11–13]. A growing body of evidence shows that sepsis patients, as well as animal models of sepsis, exhibit high levels of MCP-1, which are strongly correlated with organ dysfunction and mortality following sepsis [14–17]. A genomic deletion of MCP-1 in mice contributes to resistance against major *Leishmania* infection, whereas excessive MCP-1 expression in transgenic mice results in predisposition to infection with *Listeria monocytogenes* and *Mycobacterium tuberculosis* [18, 19]. Studies have shown that antibody neutralization or a specific antagonist of MCP-1 in mouse models of sepsis can decrease the septic response and are beneficial to survival, making MCP-1 a promising potential therapeutic target for sepsis [20–22]. Taken together, these lines of evidence indicate that continuous activation of MCP-1 plays a role in the pathogenesis and progression of sepsis.

The human MCP-1 gene is located on chromosome 17q11.2-q12 [23]. Studies have shown that two functional genetic variations within the MCP-1 gene promoter region, rs1024611 A/G and rs2857656 G/C, influence MCP-1 expression levels and result in genetic predisposition to various inflammation-related diseases [24–27]. The MCP-1 rs1024611 polymorphism within the distal regulatory region of the gene can influence the transcriptional activity of MCP-1 and contributes to susceptibility to systemic lupus erythematosus, rheumatoid arthritis and inflammatory bowel disease [27–30]. The other polymorphism, MCP-1 rs2857656, is located within the proximal promoter region of the gene and reportedly contributes to increased MCP-1 expression levels as well as increased risk of spinal tuberculosis and carotid atherosclerosis [31, 32]. However, there are currently no reports on the clinical relevance of these two MCP-1 polymorphisms to sepsis susceptibility and progression.

A growing body of work has demonstrated that many variations in genes associated with the inflammatory and immune responses contribute to the occurrence and progression of sepsis [4–7]. However, to the best of our knowledge, the clinical relevance of MCP-1 genetic polymorphisms to sepsis has not been determined, adequately. Given the evidence implicating MCP-1 in the pathomechanism and progression of sepsis, we conducted this case-control study to examine whether two MCP-1 promoter polymorphisms (rs1024611 and rs2857656) are associated with sepsis in the Han Chinese population. In addition, we determined the expression levels of MCP-1, IL-6, IL-1β and TNF-α in the study subjects to assess potential associations between these genetic variations and cytokine production.

## Materials and methods

### Study population

In the present study, 403 sepsis patients (age range 23–86 years, mean 59.2; 286 men and 117 women) were enrolled within 24 hours of admission to the intensive care unit (ICU) at the Affiliated Hospital of Guangdong Medical University (Zhanjiang, China) from December 2012 to December 2015. The diagnosis of sepsis was defined following the International Sepsis Definitions Conference [33, 34]. Those patients were excluded from this study if they were combined with preexisting cancer, ACI, HIV, blood or autoimmune diseases. The peripheral blood samples were collected within 12 hours when the diagnosis of sepsis, severe sepsis, or septic shock was established. The sepsis, severe sepsis or septic shock is the initial situation of the disease in the patients. The following clinical parameters were recorded for each patient: age, sex, dysfunctional organs, source of infection, blood microbiological cultures, and Acute Physiology and Chronic Health Evaluation (APACHE) II score [35]. As a control group, 400 healthy subjects (age range 20–83 years, mean 57.5; 271 men and 129 women) without a history of sepsis, cancer, autoimmune diseases and other inflammation-related diseases were enrolled from the Health Examination Center in this hospital at the same period time. All the studied subjects were from the Chinese Han population and were at least of eighteen years old. Written informed consent was obtained from the participants prior to their enrollment in the study. Each sepsis patient’s capacity to consent was confirmed by a family member when necessary. The STROBE Statement of this study was included in the supplementary information (S1 File). This study was approved by the Ethical Committee of the Affiliated Hospital of Guangdong Medical University (No. PJ2012134).

### DNA extraction and genotyping

Genomic DNA extraction was performed by using the TIANamp Blood DNA Kit (Tiangen Biotech Co., Ltd., Beijing, China) and stored at -80°C until use. Two MCP-1 polymorphisms rs1024611 (-2518 A>G) and rs2857656 (-362 G>C) were genotyped using the SNaPshot MultiplexKit (Applied Biosystems Co., Ltd., Foster City, CA, USA), and the primers used for amplification of PCR and extension of SNaPshot were designed with GenBank database and were as follows: rs1024611F, 5' CTCTCACGCCAGCACTGACCTC 3'; rs1024611R, 5' CCAATTAGCCCATGGTCACAGA 3'; rs2857656F, 5' TAAGCTGGCAGCGAGCCTGAC 3'; rs2857656R, 5' GCCATTAAGCCCAGACTGACCA 3'. The SNaPshot PCR reaction consisted of SNaPshot Multiplex Kitreagent (5μL), templates (4μL) and primer mix (4μl). The PCR reaction protocol was as follows: 96°C for 60s; 28 cycles of 96°C for 10s, 55°C for 5s, and 60°C for 30s; 4°C for 120s. The products were purified by 1-h incubation with 1U of shrimp alkaline phosphatase (Takara: Otsu, shiga, Japan) at 37°C and 75°C for 15 minutes. Then the purified products (0.5μL) were mixed with Lizl20 Size Standard (0.5μL) and HiDi formamide (9μL) and were incubated at 95°C for 5 minutes, and were analyzed using ABIPrism 3730XL genetic sequence analyzer (Applied Biosystems, Foster City, CA, USA) and GeneMapper 4.1 (Applied Biosystems, Carlsbad, CA, USA). Finally, 10% of the samples were randomly selected as the validation group for re-genotyping. All the samples were successfully genotyped for the two MCP-1 polymorphisms. Power analyses exhibited 98.2% power for rs1024611 and 98.2% power for rs2857656 to test a genotype relative risk at an odds ratio of 1.5 at a significance level of 0.05 in this study.

### RNA extraction and quantitative real-time PCR

We randomly selected 80 sepsis patients and 80 controls from the enrolled subjects for the peripheral blood mononuclear cells (PBMCs) isolation by using density gradient centrifugation method with LymphoprepTM (Axis-Shield PoCAS, Oslo, Norway). The 80 sepsis samples included 12 mild sepsis, 38 severe sepsis and 30 septic shock samples. Among the 160 selected subjects, 21 cases and 23 controls carried the rs1024611 AA and rs28576565 GG genotypes, and 59 cases and 57 controls carried the rs1024611 GA/GG and rs28576565 GC/CC genotypes. The genomic RNA extraction from PBMCs was performed by using the RNAprep Pure Blood Kit (Sangon Biotech, Shanghai, China) and then converted to cDNA by using the First Strand cDNA Synthesis Kit (Thermo) following the protocol of the manufacturer. Next, the MCP-1 mRNA expression was detected by quantitative real-time PCR. The Primers were designed with Primer Premier 5.0 software by Shanghai Sangon Biological Engineering as follows: MCP-1: 5' CTCGCCTCCAGCATGAAAGT 3' and 5' GGTGACTGGGGCATTGATTG 3'; GAPDH: 5' TCCTACCCCCAATGTATCCG 3' and 5' CCTTTAGTGGGCCCTCGG 3'. The quantitative real-time PCR was performed using a LightCycler480 sequence detector system (Roche Applied Science, Laval, Quebec, Canada) in the following reaction conditions: 95°C/300s, and 40 cycles of 95°C/10s, 60°C/20s and 70°C/30s. The 2^-ΔΔCT^ method was used to calculate the mRNA expression of MCP-1.

### Cytokine measurement

The peripheral blood samples were obtained from the selected 80 sepsis cases and 80 controls in a sodium heparin vacutainer tube. The plasma was collected from the peripheral blood samples by centrifugation at low speed and stored at −80°C until used. The plasma concentrations of MCP-1, IL-1β, IL-6 and TNF-α were measured by using each specific enzyme linked immunosorbent assay (ELISA) kits (TianGen Biotech, Beijing, China), according to the protocol of the manufacturer. The absorbance of samples and standards were detected at 450 nm by using a microplate reader. The minimum detectable concentrations of MCP-1, IL-1β, IL-6 and TNF-α were 0.1 ng/ml, 1 pg/ml, 1 pg/ml and 1 pg/ml, respectively.

### Statistical analyses

The measurement data were shown as the mean ± standard error of the mean (SEM) and compared using Student’s t-test. Genotype/allele distribution of each polymorphism was analyzed using Chi-squared test or Fisher’s exact test, and the false discovery rate-adjusted P-value was calculated by using the Bonferroni correction in multiple-time statistics. The Hardy-Weinberg equilibrium (HWE) was used to assess the deviation of the allele or genotype frequency. A linkage disequilibrium (LD) map was construct to determine the extent of linkage disequilibrium between genetic variations using the Haploview (version 4.2) software (Jeffrey C Barrett and Mark J Daly, Cambridge, MA, USA). All statistical analyses were performed in the SPSS version 19.0 (IBM,NY, USA), and a P value < 0.05 was considered to be statistically significant.

## Results

### Clinical characteristics

The clinical parameters of the 403 consecutive patients with sepsis who were admitted to the intensive care unit (ICU) and matched the inclusive criteria from December 2012 to December 2015, as well as the 400 healthy subjects, that were included in this study are presented in Table 1. No significant differences were observed between the sepsis and healthy subject groups with respect to gender (P = 0.323) or age (P = 0.110). The 403 sepsis patients were divided into three subgroups based on the severity of their sepsis as follows: 74 patients with mild sepsis, 191 patients with severe sepsis and 138 patients with septic shock. Respiratory tract infection (63.0%), abdominal infection (23.3%) and primary bloodstream infection (11.9%) were the main sources of infection. The main pathogens identified in this study were *Acinetobacter baumannii* (23.1%), *Escherichia coli* (10.9%), *Pseudomonas aeruginosa* (9.7%) and *Staphylococcus aureus* (8.2%). Gram-negative infections, Gram-positive infections and mixed infections accounted for approximately 33.5%, 9.9% and 11.7%, respectively. The 28-day ICU mortality rate was 25.3% in this study.

**Table 1:** Clinical characteristics of sepsis patients and healthy controls.

Variable	Sepsis (n = 403) N(%)	Control (n = 400) N(%)	P value
**Demographics**			
Age, years, mean ± SD	59.2 ± 17.1	57.5 ± 13.7	0.110
Male/female, number	286/117	271/129	0.323
**Sepsis status, n(%)**			
Mild sepsis	74(18.4)	N.A	
Severe sepsis	191(47.4)	N.A	
Septic shock	138(34.2)	N.A	
**Source of infection, n(%)**			
Respiratory tract infection	254(63.0)	N.A	
Primary bloodstream infection	48(11.9)	N.A	
Abdominal infection	94(23.3)	N.A	
Urinary tract infection	22(5.5)	N.A	
Catheter-associated infection	14(3.5)	N.A	
Brain	29(7.2)	N.A	
Others	35(8.7)	N.A	
**Infection types, n(%)**			
Gram-positive	40(9.9)	N.A	
Gram-negative	135(33.5)	N.A	
Mixed Gram-negative and -positive	47(11.7)	N.A	
Fungus	87(21.6)	N.A	
Polymicrobial	64(15.9)	N.A	
Negative blood culture	31(7.7)	N.A	
**Pathogenic bacteria, n(%)**			
Acinetobacter baumannii	93(23.1)	N.A	
Monilia albican	27(6.7)	N.A	
Yeast sample sporphyte	26(6.5)	N.A	
Aspergillus	17(4.2)	N.A	
Klebsiella pneumoniae	26(6.5)	N.A	
Pseudomonas aeruginosa	39(9.7)	N.A	
Staphylococcus aureus	33(8.2)	N.A	
Escherichia coli	44(10.9)	N.A	
Others	76(18.9)	N.A	
APACHE II score	23.8±7.1	N.A	
28-day mortality, n(%)	102(25.3)	N.A	

N.A: not applicable; APACHE II: Acute Physiology and Chronic Health Evaluation II; Continuous data are expressed as the mean ± SD

### Effects of MCP-1 gene polymorphisms on sepsis risk

**Fig 1:**
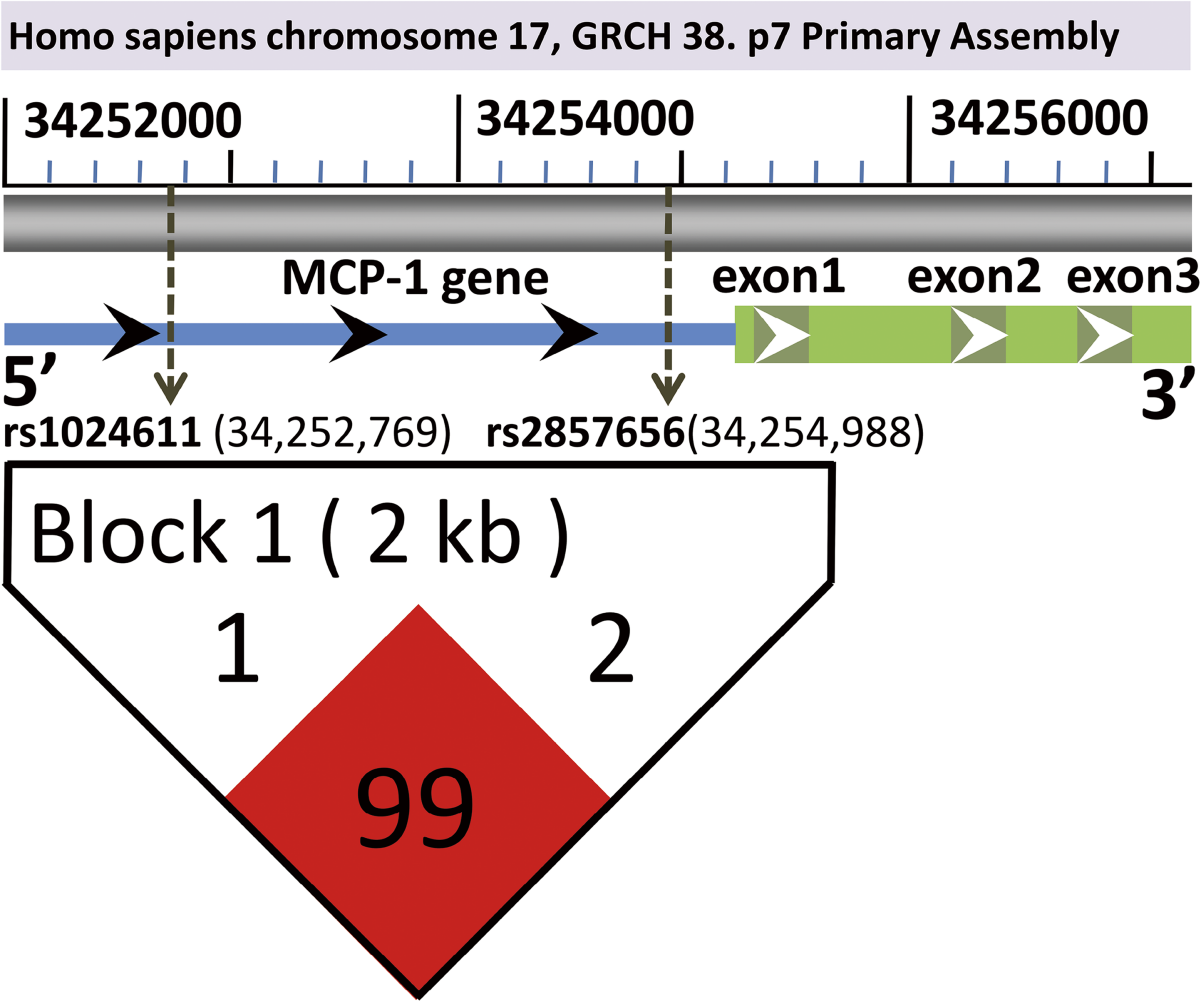
The linkage disequilibrium (LD) block (rs1024611 and rs2857565) and their locations in the promoter region of the MCP-1 gene. According to the GRCh38.p7 primary assembly, the human MCP-1 gene is located in Homo sapiens chromosome 17 (34,255,277–34,257,203). The blue bar represents the 5'-flanking region of the MCP-1 gene, and the three dark green bars individually represent its exon1, exon2 and exon3, respectively. In the visual, rs1024611 and rs2857656 are located in the upstream of the transcriptional start site (-2508 bp and -289 bp), respectively. The haplotype block (rs1024611-rs2857656, D' value = 0.995, r^2^ = 0.988) is generated using Haploview 4.2.

The genotype/allele frequency distributions of the two MCP-1 promoter polymorphisms (rs1024611 A>G and rs2857656 G>C) in the sepsis and control groups are listed in Table 2. No deviations from Hardy-Weinberg equilibrium were observed for the two MCP-1 genetic variations in the sepsis and control groups (all P>0.05, S1 Table). Significant differences between the sepsis and healthy subject groups were found for the genotype distributions of rs1024611 (P = 0.0004) and rs2857656 (P = 0.0007). The frequencies of the rs1024611 AG/GG and rs2857656 GC/CC genotypes in the sepsis group were statistically higher compared with the control group (P = 0.0001 for rs1024611: AA versus AG+GG; P = 0.0002 for rs2857656: GG versus GC+CC). The frequencies of the rs1024611 G allele and the rs2857656 C allele were overrepresented in the sepsis patients compared with the controls (P = 0.0004 for rs1024611 and P = 0.0007 for rs2857656). A haplotype block was established to determine the extent of linkage disequilibrium between polymorphisms using the Haploview software program (rs1024611-rs2857656, D' value = 0.995, r^2^ = 0.988, Fig 1). The data indicated there is a nearly complete linkage disequilibrium (LD) between the rs1024611 and rs2857656 polymorphisms.

**Table 2:** Frequencies of the MCP-1 genotypes and alleles in the sepsis patients and controls.

MCP-1	Sepsis n = 403	Control n = 400	P	P*	OR (95% CI)
**rs1024611**					
AA	78(19.4)	126(31.5)	0.0004	0.0005	-
AG	214(53.1)	186(46.5)	-	-	-
GG	111(27.5)	88(22.0)	-	-	-
AA/AG	292(72.5)	312(78.0)	0.069	0.069	0.742(0.538, 1.024)
AG/GG	325(80.6)	274(68.5)	0.0001	0.0004	1.916(1.384, 2.652)
A	370(45.9)	438(54.8)	-	-	1.000 (reference)
G	436(54.1)	362(45.2)	0.0004	0.0005	1.426(1.171, 1.735)
**rs2857656**					
GG	79(19.6)	125(31.3)	0.0007	0.0009	-
GC	213(52.9)	186(46.5)	-	-	-
CC	111(27.5)	89(22.2)	-	-	-
GG/GC	292(72.5)	311(77.8)	0.083	0.083	0.753(0.546, 1.038)
GC/CC	324(80.4)	275(68.8)	0.0002	0.0008	1.864(1.348, 2.579)
G	371(46.0)	436(54.5)	-	-	1.000 (reference)
C	435(54.0)	364(45.5)	0.0007	0.0009	1.404(1.154, 1.709)

OR: odds ratio; 95% CI: 95% confidence interval

* False discovery rate-adjusted P-value for multiple hypotheses testing using the Benjamin-Hochberg method.

### Distributions of MCP-1 allele and genotype frequencies in the sepsis subgroups

We further divided the 403 cases into three subgroups based on the severity of sepsis to evaluate potential associations between MCP-1 genetic variations and sepsis progression. As presented in Table 3, the genotype distributions of the two polymorphisms in the subgroup of mild sepsis were significantly different from those in the severe sepsis (P = 0.0005 for rs1024611 and rs2857656) and septic shock (P = 0.010 for rs1024611, and P = 0.015 for rs2857656) subgroups. The frequencies of both the rs1024611 G and rs2857656 C alleles were observed to be overrepresented in the severe sepsis/septic shock subgroups compared with the mild sepsis subgroup, suggesting a role for rs1024611 A>G and rs2857656 G>C in the progression of mild sepsis to severe sepsis/septic shock.

**Table 3:** Genotype and allele frequencies distribution in the different sepsis status.

MCP-1	Mild sepsis n = 74 (%)	Severe sepsis n = 191 (%)	Septic shock n = 138 (%)	P1	P2	P1*	P2*
**rs1024611**							
AA	25(33.8)	28(14.7)	25(18.1)	0.0005	0.010	0.001	0.020
AG/GG	49(66.2)	163(85.3)	113(81.9)				
A	83(56.1)	163(42.7)	124(44.9)	0.0055	0.029	0.0055	0.029
G	65(43.9)	219(57.3)	152(55.1)				
**rs2857656**							
GG	25(33.8)	28(14.7)	26(18.8)	0.0005	0.015	0.001	0.030
GC/CC	49(66.2)	163(85.3)	112(81.2)				
G	83(56.1)	163(42.7)	125(45.3)	0.0055	0.034	0.0055	0.034
C	65(43.9)	219(57.3)	151(54.7)				

P1: mild sepsis group versus severe sepsis; P2: mild sepsis group versus septic shock.

*False discovery rate-adjusted P-value for multiple hypotheses testing using the Benjamin-Hochberg method.

### Effects of MCP-1 gene polymorphisms on the expression of MCP-1

**Fig 2:**
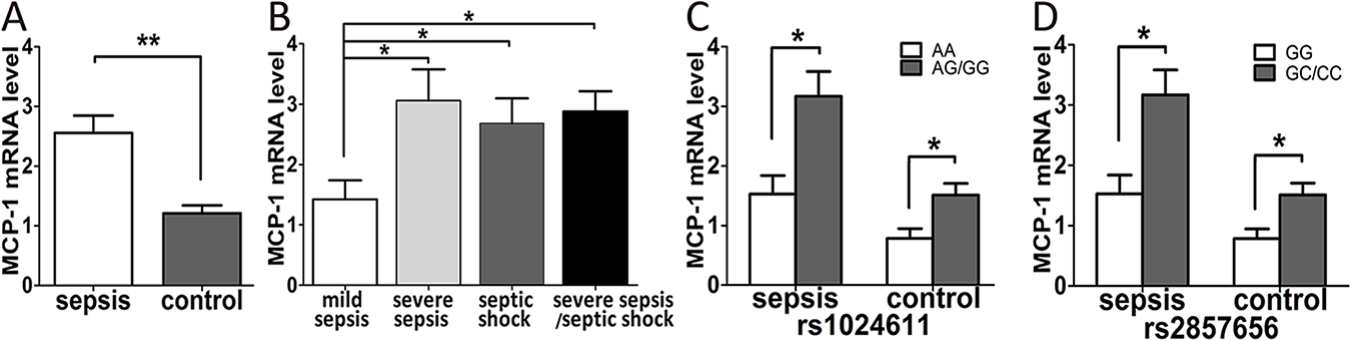
Real-time PCR analysis of the monocyte chemo-attractant protein 1 (MCP-1) mRNA expression levels in sepsis cases (n = 80) and healthy controls (n = 80). Expression levels of MCP-1 in sepsis patients and healthy controls (A). Expression levels of MCP-1 in mild sepsis, severe sepsis and septic shock subgroups (B). The distribution of MCP-1 mRNA expression levels in groups of sepsis patients with different rs1024611 genotypes (C) and different rs2857656 genotypes (D). The horizontal line stands for the median expression level with each group. * P <0.05; ** P <0.01; *** P <0.001.

**Fig 3:**
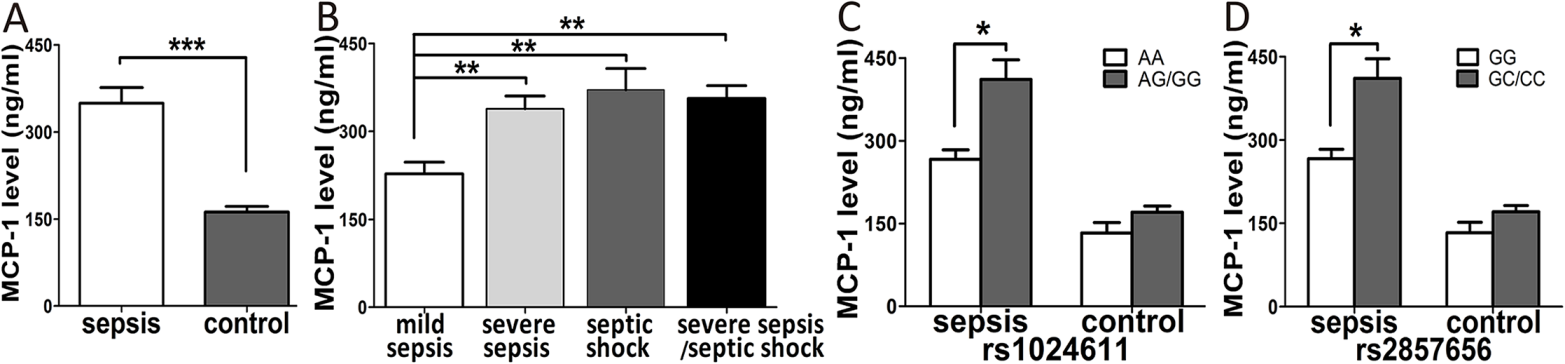
The plasma concentration of MCP-1 in sepsis patients (n = 80) and healthy controls (n = 80). The plasma concentration of MCP-1 in sepsis patients and healthy controls (A), and the plasma concentration of MCP-1 in mild sepsis, severe sepsis and septic shock subgroups (B). The distribution of the plasma concentration of MCP-1 in groups of sepsis patients with different rs1024611 genotypes (C) and different rs2857656 genotypes (D). The horizontal line stands for the median concentration with each group. * P <0.05; ** P <0.01; *** P <0.001.

In total, 80 sepsis cases and 80 healthy subjects were randomly selected to investigate MCP-1 mRNA expression in peripheral blood mononuclear cells. Consistent with several previous studies [16, 17], expression levels of the MCP-1 mRNA in the sepsis group were significantly higher than in the control group (P = 0.002, Fig 2A). Among the three sepsis subgroups, MCP-1 mRNA expression levels in the severe sepsis/septic shock subgroups were statistically higher compared with the mild sepsis subgroup (P<0.05, Fig 2B). We further assessed the influence of MCP-1 genetic variations on MCP-1 mRNA expression in the sepsis and control groups. Significantly higher expression levels of the MCP-1 mRNA were found in sepsis patients and healthy controls carrying the rs1024611 AG/GG genotypes or rs2857656 GC/CC genotypes (Fig 2C and 2D). In addition, we determined the plasma concentrations of the MCP-1 protein in sepsis and control groups, and our results were consistent with the MCP-1 mRNA expression levels, whereas MCP-1 plasma concentrations were normal in healthy controls with these genotypes (Fig 3).

### Effects of MCP-1 gene polymorphisms on the plasma concentrations of related pro-inflammatory cytokines

**Fig 4:**
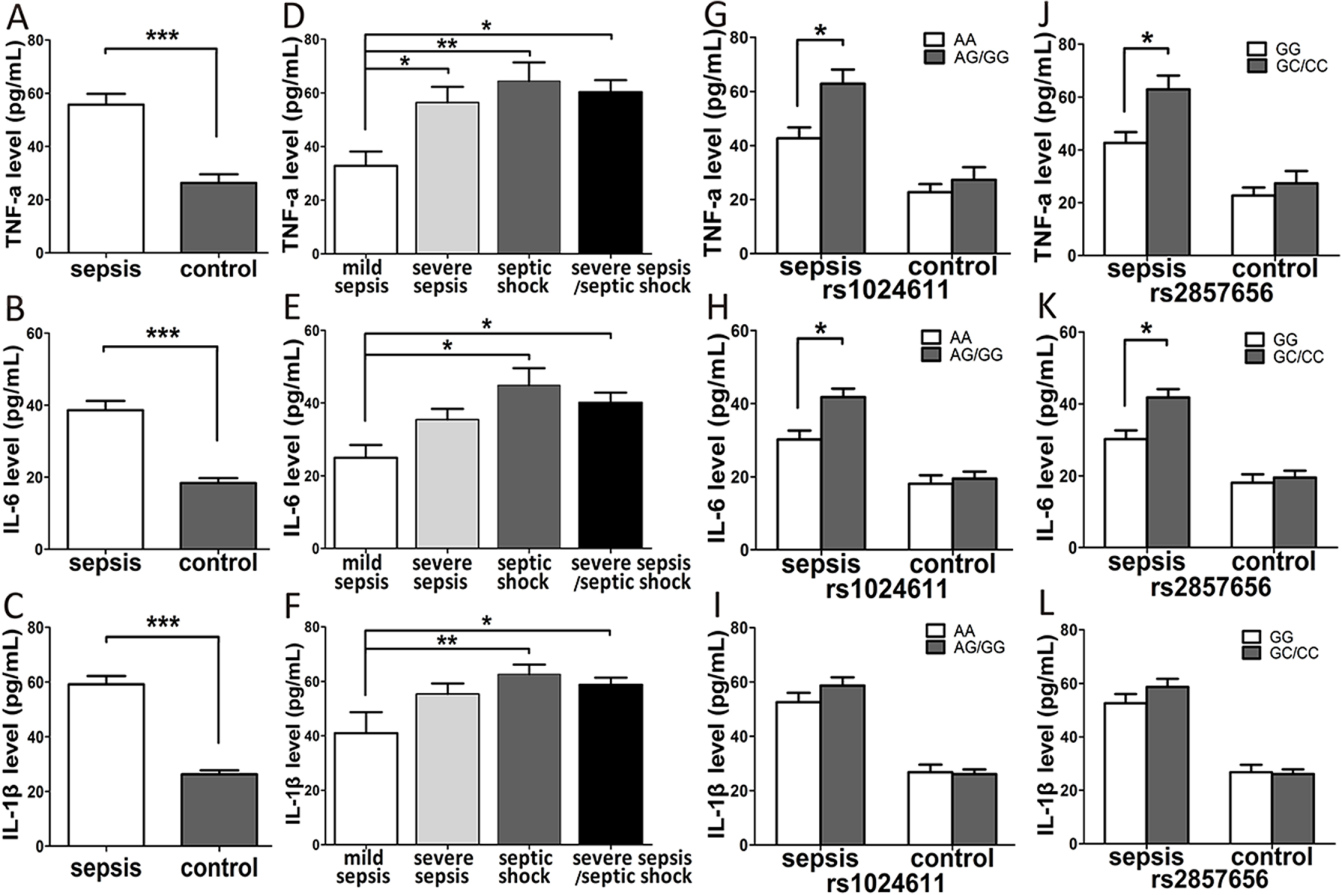
The plasma concentrations of pro-inflammatory cytokines polymorphisms in sepsis patients (n = 80) and healthy controls (n = 80). The plasma concentration of TNF-α (A), IL-6 (B) and IL-1β (C) in sepsis cases (n = 80) and controls (n = 80), and the plasma concentration of TNF-α (D), IL-6 (E) and IL-1β (F) in mild sepsis, severe sepsis and septic shock subgroups. The distribution of the plasma concentration of TNF-α (G), IL-6 (H) and IL-1β (I) in groups of sepsis patients with different rs1024611 genotypes. The distribution of the plasma concentration of TNF-α (J), IL-6 (K) and IL-1β (L) in groups of sepsis patients with different rs2857656 genotypes. The horizontal line stands for the median concentration with each group. * P <0.05; ** P <0.01; *** P <0.001.

We determined the plasma concentrations of TNF-α, IL-6 and IL-1β to determine whether MCP-1 gene polymorphisms had any effect on the production of these related cytokines in the sepsis and control groups. The sepsis patients exhibited significantly higher plasma concentrations of TNF-α, IL-6 and IL-1β compared with the healthy controls (Fig 4A, 4B and 4C). Among the three sepsis subgroups, the plasma concentrations of TNF-α, IL-6 and IL-1β in the severe sepsis/septic shock subgroups were significantly higher compared with the mild sepsis subgroup (Fig 4D, 4E and 4F). Furthermore, the MCP-1 rs1024611 AG/GG and rs2857656 GC/CC genotype carriers exhibited significantly higher concentrations of TNF-α and IL-6 compared with the rs1024611 AA and rs2857656 GG genotype carriers among the sepsis patients (Fig 4G, 4H, 4J and 4K). However, no significant differences in the IL-1β concentrations were observed among the different genotypes in sepsis cases or healthy controls (Fig 4I and 4L).

## Discussion

To our knowledge, this study was the first to explore the clinical relevance of two specific MCP-1 gene promoter polymorphisms, rs1024611 (-2518 A>G) and rs2857656 (-362 G>C), for sepsis susceptibility in the Chinese population. Our results showed that the AG/GG genotypes at rs1024611 and GC/CC genotypes at rs2857656 were associated with susceptibility to sepsis. Furthermore, we identified additional stratifications indicating that the rs1024611 G allele and rs2857656 C allele were both overrepresented among the severe sepsis/septic shock subgroups compared with the mild sepsis subgroup, suggesting a possible role for rs1024611 A>G and rs2857656 G>C in promoting sepsis progression.

Accumulating evidence indicates that MCP-1 plays an important role in the pathogenic mechanisms leading to sepsis [14–17]. MCP-1 is a member of the CC chemokine family, and it is an important molecule for monocytes recruitment under acute inflammatory conditions and endothelial activation by regulating inflammation progression through the production of pro-inflammatory cytokines [36, 37]. Several studies have shown that the expression levels of MCP-1 are markedly increased in various murine models of sepsis, which reflect the organ dysfunction and mortality seen in sepsis patients [15, 38]. Consistent with previous studies [16, 17], our results showed that MCP-1 expression levels in the sepsis group were significantly higher than in the control group, and expression levels also increased with severity of sepsis. These results confirm an important role for MCP-1 in the pathomechanisms and progression of sepsis as a pro-inflammatory mediator, and they also suggest that MCP-1 could be used as an indicator of sepsis severity.

Recently, several studies indicated that MCP-1 genetic variations within the regulatory regions of the gene could predispose patients to certain inflammation-related diseases by altering MCP-1 expression levels or certain linkage correlations under conditions of infection or systemic inflammatory response [25–27]. MCP-1 transcription is under the control of two distinct regions in the 5'-flanking region of the MCP-1 gene [39]. The -2518 A/G (rs1024611) promoter polymorphism influences the distal regulatory region, which is located upstream of the transcription start site (1.9–2.7 kb) [40], and is considered a good candidate for genetic predisposition to various inflammatory diseases, such as Crohn's disease [41], spontaneous bacterial peritonitis [24] and systemic lupus erythematosus [28]. Another proximal regulatory region located upstream of the transcriptional start site (>150 bases) appears to contain potential transcription factor binding sites [42]. The MCP-1 rs2857656 polymorphism located in this proximal promoter region was reported to increase the risk of carotid atherosclerosis by enhancing transcriptional activity of the MCP-1 gene [43]. Consequently, we conducted this study to assess the roles of these two MCP-1 promoter polymorphisms in the susceptibility to and development of sepsis. Our data show that the sepsis patients carrying the rs1024611 AG/GG or rs2857656 GC/CC genotypes presented with higher MCP-1 expression levels compared with the carriers of the rs1024611 AA or rs2857656 GG genotypes. These results suggest that these two SNPs are functional polymorphisms that upregulate the expression levels of MCP-1 through enhanced transcription, ultimately promoting MCP-1-mediated inflammatory progression and resulting in a predisposition towards sepsis. In addition, the frequencies of the rs1024611 G allele and rs2857656 C allele were both overrepresented among the severe sepsis/septic shock subgroups compared with the mild sepsis subgroup, further supporting the idea that rs12692386 (-2518 A>G) and rs2857656 (-362 G>C) are statistically significant prognostic factors that acts as genetic indicators of the sepsis risk and development. It is worth nothing that, as well as the closely inflammation-related disease, the recent GWAS study related the inflammatory bowel disease (IBD) identified rs3091315 and rs3091316 in the region as the susceptibility SNPs [44, 45], which there is strong linkage disequilibrium among rs1024611 and rs2857656, rs3091315 and rs3091316 (r^2^>0.9) in each continental population, supporting our report on the association between these two SNPs and sepsis risk. Future studies will investigate the molecular mechanisms affected by these two functional polymorphisms using promoter prediction techniques and a cellular sepsis model for experimental verification of these findings.

MCP-1 plays pivotal roles in modulating monocyte chemotaxis and endothelial activation as well as regulation of inflammatory progression and the production of pro-inflammatory cytokines [17, 46, 47]. Accumulating evidence demonstrates that the inhibition or specific antagonism of MCP-1 results in decreased release of TNF-α, IL-1β and IL-6 by macrophages and confers survival benefits on mice following sepsis [20, 21, 48, 49]. Moreover, a recent study suggested that the modulation of monocyte recruitment and endothelial activation during LPS-induced endotoxemia are mediated by MCP-1 [50], and another study by Katherine et al. showed that antibody neutralization of MCP-1 in a mouse model of sepsis leads to greatly decreased transcription of IL-1α, IL-1β, and IL-6 in the diaphragm [51]. We found that plasma concentrations of TNF-α, IL-1β and IL-6 in the sepsis group were significantly higher than in the healthy control group, and the levels of these cytokines also increased with sepsis severity. Importantly, the plasma concentrations of TNF-α and IL-6 were significantly increased in sepsis patients with rs1024611 AG/GG or rs2857656 GC/CC genotypes, accompanied by an upregulation of MCP-1. These results are consistent with previous studies involving models of sepsis where the blockage of MCP-1 inhibited the expression of pro-inflammatory cytokines and conferred a survival benefit to mice following sepsis [20–22]. We inferred that rs1024611 A>G and rs2857656 G>C upregulate MCP-1 expression levels by enhancing transcriptional activity of the MCP-1 gene, thereby causing excessive macrophage activation, increased production of pro-inflammatory cytokines and ultimately a predisposition to and development of sepsis.

Several important limitations should be acknowledged in this study. First, the limited number of patients in the study could have affected estimations of our preliminary conclusions. Second, only a few MCP-1 genetic variations implicated in the susceptibility and progression of sepsis were studied, and it is possible that other MCP-1 polymorphisms associated with sepsis remain to be identified. Third, the subjects enrolled in this study were only from the Han Chinese ethnic group. Thus, further biological studies with a larger sample size of sepsis patients from different ethnicities will be necessary to verify the association of MCP-1 polymorphisms with sepsis. Moreover, not only MCP-1 (CCL2) but also other chemokine genes, such as CCL7 and CCL11, are located near to the positions of the rs1024611 and rs2857656 polymorphisms, so it is also possible that these polymorphisms may affects the other activity of neighboring genes.

In conclusion, this study was the first to demonstrate an association between two MCP-1 genetic variations (rs1024611 G and rs2857656 C allele/the AG haplotype) that are associated with predisposition to and protection against sepsis, respectively. Moreover, the high-risk rs1024611 AG/GG and rs2857656 GC/CC genotypes affected the transcriptional activity and expression of MCP-1, accompanied by the upregulation of pro-inflammation cytokines. These findings suggest that these two MCP-1 promoter polymorphisms are clinically significant and further validate the importance of MCP-1 as a therapeutic target in the pathogenesis and progression of sepsis.

## Supporting information

S1 File STROBE Statement-checklist of items that should be included in reports of observational studies. (PDF)

S1 Table The Hardy-Weinberg equilibrium of the two MCP-1 promoter polymorphisms. (PDF)

## References

[1] Hotchkiss RS, Sherwood ER. Immunology. Getting sepsis therapy right. Science. 2015; 347:1201–1202. doi: 10.1126/science.aaa8334 25766219 10.1126/science.aaa8334 PMC4398343

[2] Szakmany T, Lundin RM, Sharif B, Ellis G, Morgan P, Kopczynska M, et al Sepsis Prevalence and Outcome on the General Wards and Emergency Departments in Wales: Results of a Multi-Centre, Observational, Point Prevalence Study. PLoS One. 2016;11:e0167230 doi: 10.1371/journal.pone.0167230 27907062 10.1371/journal.pone.0167230 PMC5132245

[3] Suffredini AF, Munford RS. Novel therapies for septic shock over the past 4 decades. JAMA. 2011;306:194–199. doi: 10.1001/jama.2011.909 21750297 10.1001/jama.2011.909

[4] Shao Y, Shao X, He J, Cai Y, Zhao J, Chen F, et al The promoter polymorphisms of receptor for advanced glycation end products were associated with the susceptibility and progression of sepsis. Clin Genet. 2017;91:564–575. doi: 10.1111/cge.12800 27172264 10.1111/cge.12800

[5] Cui L, Gao Y, Xie Y, Wang Y, Cai Y, Shao X, et al An ADAM10 promoter polymorphism is a functional variant in severe sepsis patients and confers susceptibility to the development of sepsis. Crit Care. 2015;19:73 doi: 10.1186/s13054-015-0796-x 25888255 10.1186/s13054-015-0796-x PMC4373036

[6] Kimura T, Watanabe E, Sakamoto T, Takasu O, Ikeda T, Ikeda K, et al Autophagy-related IRGM polymorphism is associated with mortality of patients with severe sepsis. PLoS One. 2014;9:e91522 doi: 10.1371/journal.pone.0091522 24626347 10.1371/journal.pone.0091522 PMC3953488

[7] Shao Y, He J, Chen F, Cai Y, Zhao J, Lin Y, et al Association Study Between Promoter Polymorphisms of ADAM17 and Progression of Sepsis. Cell Physiol Biochem. 2016;39:1247–1261. doi: 10.1159/000447830 27607600 10.1159/000447830

[8] Namath A, Patterson AJ. Genetic polymorphisms in sepsis. Crit Care Nurs Clin North Am. 2011;23:181–202. doi: 10.1016/j.ccell.2010.12.011 21316575 10.1016/j.ccell.2010.12.011

[9] Shimada T, Oda S, Sadahiro T, Nakamura M, Hirayama Y, Watanabe E, et al Outcome prediction in sepsis combined use of genetic polymorphisms—A study in Japanese population. Cytokine. 2011;54:79–84. doi: 10.1016/j.cyto.2010.12.001 21282064 10.1016/j.cyto.2010.12.001

[10] Sipahi T, Pocan H, Akar N. Effect of various genetic polymorphisms on the incidence and outcome of severe sepsis. Clin Appl Thromb Hemost. 2006;12:47–54. 16444434 10.1177/107602960601200108

[11] Deshmane SL, Kremlev S, Amini S, Sawaya BE. Monocyte chemoattractant protein-1 (MCP-1): an overview. J Interferon Cytokine Res. 2009;29:313–326. doi: 10.1089/jir.2008.0027 19441883 10.1089/jir.2008.0027 PMC2755091

[12] Gerszten RE, Garcia-Zepeda EA, Lim YC, Yoshida M, Ding HA, Gimbrone MA Jr., et al MCP-1 and IL-8 trigger firm adhesion of monocytes to vascular endothelium under flow conditions. Nature. 1999;398:718–723. doi: 10.1038/19546 10227295 10.1038/19546

[13] Ziraldo C, Vodovotz Y, Namas RA, Almahmoud K, Tapias V, Mi Q, et al Central role for MCP-1/CCL2 in injury-induced inflammation revealed by in vitro, in silico, and clinical studies. PLoS One. 2013;8:e79804 doi: 10.1371/journal.pone.0079804 24312451 10.1371/journal.pone.0079804 PMC3849193

[14] Bossink AW, Paemen L, Jansen PM, Hack CE, Thijs LG, Van Damme J. Plasma levels of the chemokines monocyte chemotactic proteins-1 and -2 are elevated in human sepsis. Blood. 1995;86:3841–3847. 7579352

[15] Bozza FA, Salluh JI, Japiassu AM, Soares M, Assis EF, Gomes RN, et al Cytokine profiles as markers of disease severity in sepsis: a multiplex analysis. Crit Care. 2007;11:R49 doi: 10.1186/cc5783 17448250 10.1186/cc5783 PMC2206478

[16] Matsukawa A, Hogaboam CM, Lukacs NW, Lincoln PM, Strieter RM, Kunkel SL. Endogenous MCP-1 influences systemic cytokine balance in a murine model of acute septic peritonitis. Exp Mol Pathol. 2000;68:77–84. doi: 10.1006/exmp.1999.2296 10716911 10.1006/exmp.1999.2296

[17] Tsuda Y, Takahashi H, Kobayashi M, Hanafusa T, Herndon DN, Suzuki F. CCL2, a product of mice early after systemic inflammatory response syndrome (SIRS), induces alternatively activated macrophages capable of impairing antibacterial resistance of SIRS mice. J Leukoc Biol. 2004;76:368–373. doi: 10.1189/jlb.1203645 15123772 10.1189/jlb.1203645

[18] Gu L, Tseng S, Horner RM, Tam C, Loda M, Rollins BJ. Control of TH2 polarization by the chemokine monocyte chemoattractant protein-1. Nature. 2000;404:407–411. doi: 10.1038/35006097 10746730 10.1038/35006097

[19] Lu B, Rutledge BJ, Gu L, Fiorillo J, Lukacs NW, Kunkel SL, et al Abnormalities in monocyte recruitment and cytokine expression in monocyte chemoattractant protein 1-deficient mice. J Exp Med. 1998;187:601–608. 9463410 10.1084/jem.187.4.601 PMC2212142

[20] Ramnath RD, Ng SW, Guglielmotti A, Bhatia M. Role of MCP-1 in endotoxemia and sepsis. Int Immunopharmacol. 10.1016/j.intimp.2008.01.033 18442784

[21] Speyer CL, Gao H, Rancilio NJ, Neff TA, Huffnagle GB, Sarma JV, et al Novel chemokine responsiveness and mobilization of neutrophils during sepsis. Am J Pathol. 2004;165:2187–2196. doi: 10.1016/S0002-9440(10)63268-3 15579460 10.1016/S0002-9440(10)63268-3 PMC1618724

[22] Taub DD. Chemokine-leukocyte interactions. The voodoo that they do so well. Cytokine Growth Factor Rev. 1996;7:355–376. 9023058 10.1016/s1359-6101(97)89237-4

[23] Da LS, Zhang Y, Zhang S, Qian YC, Zhang Q, Jiang F, et al Association between MCP-1 -2518A/G polymorphism and cancer risk: evidence from 19 case-control studies. PLoS One. 2013;8:e82855 doi: 10.1371/journal.pone.0082855 24367564 10.1371/journal.pone.0082855 PMC3867394

[24] Salama MK, Sabry D, Al-Ghussein MA, Ahmed R, AbdAllah S, Taha FM, et al Molecular detection of monocyte chemotactic protein-1 polymorphism in spontaneous bacterial peritonitis patients. World J Gastroenterol. 2014;20:11793–11799. doi: 10.3748/wjg.v20.i33.11793 25206284 10.3748/wjg.v20.i33.11793 PMC4155370

[25] Thye T, Nejentsev S, Intemann CD, Browne EN, Chinbuah MA, Gyapong J, et al MCP-1 promoter variant -362C associated with protection from pulmonary tuberculosis in Ghana, West Africa. Hum Mol Genet. 2009;18:381–388. doi: 10.1093/hmg/ddn352 18940815 10.1093/hmg/ddn352 PMC2638774

[26] Walczak A, Przybylowska K, Sygut A, Dziki L, Chojnacki C, Chojnacki J, et al The -2518 A/G MCP-1 polymorphism as a risk factor of inflammatory bowel disease. Pol Przegl Chir. 2012;84:238–241. doi: 10.2478/v10035-012-0039-7 22763298 10.2478/v10035-012-0039-7

[27] Rovin BH, Lu L, Saxena R. A novel polymorphism in the MCP-1 gene regulatory region that influences MCP-1 expression. Biochem Biophys Res Commun. 1999;259:344–348. doi: 10.1006/bbrc.1999.0796 10362511 10.1006/bbrc.1999.0796

[28] Aguilar F, Gonzalez-Escribano MF, Sanchez-Roman J, Nunez-Roldan A. MCP-1 promoter polymorphism in Spanish patients with systemic lupus erythematosus. Tissue Antigens. 2001;58:335–338. 11844145 10.1034/j.1399-0039.2001.580508.x

[29] Li YW, Yang CQ, Xiao YL, Li J, Xie CX, Zhang SH, et al The -A2518G polymorphism in the MCP-1 gene and inflammatory bowel disease risk: A meta-analysis. J Dig Dis. 2015;16:177–185. doi: 10.1111/1751-2980.12232 25620350 10.1111/1751-2980.12232

[30] Ozyurek AR, Gurses D, Ulger Z, Levent E, Bakiler AR, Berdeli A. Allelic frequency of the MCP-1 promoter -2518 polymorphism in the Turkish population and in Turkish patients with juvenile rheumatoid arthritis. Clin Rheumatol. 2007;26:546–550. doi: 10.1007/s10067-006-0347-6 16835702 10.1007/s10067-006-0347-6

[31] Guo C, Zhang H, Gao Q, He D, Tang M, Liu S, et al Monocyte chemoattractant protein-1 in spinal tuberculosis: -362G/C genetic variant and protein levels in Chinese patients. Diagn Microbiol Infect Dis. 2014;78:49–52. doi: 10.1016/j.diagmicrobio.2013.07.024 24183600 10.1016/j.diagmicrobio.2013.07.024

[32] Nyquist P, Zhang J, De Graba TJ. The -928 G/C and -362 G/C single-nucleotide polymorphisms in the promoter of MCP-1: Increased transcriptional activity and novel binding sites. Cerebrovasc Dis. 2010;29:242–247. doi: 10.1159/000267849 20029197 10.1159/000267849

[33] Dellinger RP, Levy MM, Rhodes A, Annane D, Gerlach H, Opal SM, et al Surviving Sepsis Campaign: international guidelines for management of severe sepsis and septic shock, 2012. Intensive Care Med. 2013;39:165–228. doi: 10.1007/s00134-012-2769-8 23361625 10.1007/s00134-012-2769-8 PMC7095153

[34] Singer M, Deutschman CS, Seymour CW, Shankar-Hari M, Annane D, Bauer M, et al The Third International Consensus Definitions for Sepsis and Septic Shock (Sepsis-3). JAMA. 2016;315:801–810. doi: 10.1001/jama.2016.0287 26903338 10.1001/jama.2016.0287 PMC4968574

[35] Knaus WA, Draper EA, Wagner DP, Zimmerman JE. APACHE II: a severity of disease classification system. Crit Care Med. 1985;13:818–829. 3928249

[36] Fong DS, Aiello LP, Ferris FL, 3rd, Klein R. Diabetic retinopathy. Diabetes Care. 2004;27:2540–2553. 15451934 10.2337/diacare.27.10.2540

[37] Holtkamp GM, De Vos AF, Peek R, Kijlsta A. Analysis of the secretion pattern of monocyte chemotactic protein-1 (MCP-1) and transforming growth factor-beta 2 (TGF-beta2) by human retinal pigment epithelial cells. Clin Exp Immunol. 1999;118:35–40. doi: 10.1046/j.1365-2249.1999.01016.x 10540157 10.1046/j.1365-2249.1999.01016.x PMC1905390

[38] Zhang H, Zhi L, Moochhala S, Moore PK, Bhatia M. Hydrogen sulfide acts as an inflammatory mediator in cecal ligation and puncture-induced sepsis in mice by upregulating the production of cytokines and chemokines via NF-kappaB. Am J Physiol Lung Cell Mol Physiol. 2007;292:L960–971. doi: 10.1152/ajplung.00388.2006 17209138 10.1152/ajplung.00388.2006

[39] Ueda A, Okuda K, Ohno S, Shirai A, Igarashi T, Matsunaga K, et al NF-kappa B and Sp1 regulate transcription of the human monocyte chemoattractant protein-1 gene. J Immunol. 1994;153:2052–2063. 8051410

[40] Ping D, Jones PL, Boss JM. TNF regulates the in vivo occupancy of both distal and proximal regulatory regions of the MCP-1/JE gene. Immunity. 1996;4:455–469. 8630731 10.1016/s1074-7613(00)80412-4

[41] Palmieri O, Latiano A, Salvatori E, Valvano MR, Bossa F, Latiano T, et al The -A2518G polymorphism of monocyte chemoattractant protein-1 is associated with Crohn's disease. Am J Gastroenterol. 2010;105:1586–1594. doi: 10.1038/ajg.2010.4 20125127 10.1038/ajg.2010.4

[42] Valente AJ, Xie JF, Abramova MA, Wenzel UO, Abboud HE, Graves DT. A complex element regulates IFN-gamma-stimulated monocyte chemoattractant protein-1 gene transcription. J Immunol. 1998;161:3719–3728. 9759897

[43] Nyquist PA, Winkler CA, McKenzie LM, Yanek LR, Becker LC, Becker DM. Single nucleotide polymorphisms in monocyte chemoattractant protein-1 and its receptor act synergistically to increase the risk of carotid atherosclerosis. Cerebrovasc Dis. 2009;28:124–130. doi: 10.1159/000223437 19506371 10.1159/000223437 PMC2914430

[44] Franke A, McGovern DP, Barrett JC, Wang K, Radford-Smith GL, Ahmad T, et al Genome-wide meta-analysis increases to 71 the number of confirmed Crohn's disease susceptibility loci. Nat Genet. 2010;42:1118–25. doi: 10.1038/ng.717 21102463 10.1038/ng.717 PMC3299551

[45] Jostins L, Ripke S, Weersma RK, Duerr RH, McGovern DP, Hui KY, et al Host-microbe interactions have shaped the genetic architecture of inflammatory bowel disease. Nature. 2012;491:119–24. doi: 10.1038/nature11582 23128233 10.1038/nature11582 PMC3491803

[46] Han YL, Li YL, Jia LX, Cheng JZ, Qi YF, Zhang HJ, et al Reciprocal interaction between macrophages and T cells stimulates IFN-gamma and MCP-1 production in Ang II-induced cardiac inflammation and fibrosis. PLoS One. 2012;7:e35506 doi: 10.1371/journal.pone.0035506 22567105 10.1371/journal.pone.0035506 PMC3342394

[47] Hanemann AL, Liborio AB, Daher EF, Martins AM, Pinheiro MC, Sousa MS, et al Monocyte chemotactic protein-1 (MCP-1) in patients with chronic schistosomiasis mansoni: evidences of subclinical renal inflammation. PLoS One. 2013;8:e80421 doi: 10.1371/journal.pone.0080421 24265821 10.1371/journal.pone.0080421 PMC3827226

[48] Damas JK, Aukrust P, Ueland T, Odegaard A, Eiken HG, Gullestad L, et al Monocyte chemoattractant protein-1 enhances and interleukin-10 suppresses the production of inflammatory cytokines in adult rat cardiomyocytes. Basic Res Cardiol. 2001;96:345–352. 11518190 10.1007/s003950170042

[49] Slimani H, Zhai Y, Yousif NG, Ao L, Zeng Q, Fullerton DA, et al Enhanced monocyte chemoattractant protein-1 production in aging mice exaggerates cardiac depression during endotoxemia. Crit Care. 2014;18:527 doi: 10.1186/s13054-014-0527-8 25209241 10.1186/s13054-014-0527-8 PMC4172828

[50] Turler A, Schwarz NT, Turler E, Kalff JC, Bauer AJ. MCP-1 causes leukocyte recruitment and subsequently endotoxemic ileus in rat. Am J Physiol Gastrointest Liver Physiol. 2002;282:G145–155. doi: 10.1152/ajpgi.00263.2001 11751168 10.1152/ajpgi.00263.2001

[51] Labbe K, Danialou G, Gvozdic D, Demoule A, Divangahi M, Boyd JH, et al Inhibition of monocyte chemoattractant protein-1 prevents diaphragmatic inflammation and maintains contractile function during endotoxemia. Crit Care. 2010;14:R187 doi: 10.1186/cc9295 20950459 10.1186/cc9295 PMC3219293

