# Supplementary material for: Association study of MCP-1 promoter polymorphisms with the susceptibility and progression of sepsis
Source: PLoS One. 2017 May 4;12(5):e0176781. doi: 10.1371/journal.pone.0176781 (PMC5417587; doi:10.1371/journal.pone.0176781)
Supplement: S1 Table — (PDF) [file pone.0176781.s002.pdf]

S1 Table.

Hardy-Weinberg P	sepsis(n=403)	control(n=400)
rs1024611	0.165	0.218
rs2857656	0.200	0.212
